# Comparing anti-tau antibodies under clinical trials and their epitopes on tau pathologies

**DOI:** 10.1186/s13024-024-00769-x

**Published:** 2024-10-19

**Authors:** Ha-Lim Song, Min-Seok Kim, Woo-Young Cho, Ye-Seul Yoo, Jae-You Kim, Tae-Wook Kim, Hyori Kim, Dong-Hou Kim, Seung-Yong Yoon

**Affiliations:** 1 ADEL Institute of Science & Technology (AIST), ADEL, Inc, Seoul, Korea; 2 Department of Brain Science, Asan Medical Center, University of Ulsan College of Medicine, Brain Korea 21 project, Seoul, Korea; 3 Convergence Medicine Research Center, Asan Institute for Life Sciences, Asan Medical Center, Seoul, South Korea; 4 Stem Cell Immunomodulation Research Center (SCIRC), University of Ulsan College of Medicine, Seoul, Korea

To the Editor,

Tauopathies, including Alzheimer’s disease (AD), are characterized by the accumulation of abnormal tau protein deposits in the brain. Tau exists in multiple heterogenous forms of various polypeptide fragments by enzymatic cleavage and post-translational modifications (PTMs) [1]. Insights from clinical trials of anti-β-amyloid (Aβ) antibodies highlight the importance of epitope selection, as targeting Aβ protofibrils or N-terminus influenced both target engagement and downstream pathogenic processes [2]. Initially, anti-tau antibodies targeting the N-terminus were developed because these N-terminal fragments predominated in AD cerebrospinal fluid (CSF) and were implicated in tau spread [3]. However, these trials ultimately failed [4], aligning with earlier findings that indicated insufficient inhibition of tau seeding [5]. Although other epitopes, such as mid-region, microtubule-binding region (MTBR) and C-terminus, are being explored, the most effective target remains unclear. Certain tau fragments are suggested to play critical roles in tau pathology development [1] and studies in the interstitial fluid (ISF) of tau transgenic mice brains show that secreted tau is primarily truncated during disease progression [6]. The complexity of tau cleavage and PTMs emphasizes the significance of epitope selection, especially in the context of low brain penetration of antibodies, to effectively bind seed-competent forms and counteract propagation.

To investigate this issue, the potency of various anti-tau antibodies under clinical trials was compared using sarkosyl-insoluble fractions isolated from AD patient brains. Inhibition of tau seeding by antibodies targeting the N-terminus (antibody A), mid-region (antibody B), and MTBR (antibody C and D) (Fig. 1a and table S1) was tested using tau fluorescence resonance energy transfer (FRET) cells. Initial study using fraction from a single patient to determine adequate concentration yielded dose-dependent inhibition of tau seeding with anti-tau antibody treatment. Cells treated with anti-acetylated lysine-280 (acK280) antibody, antibody C, showed the most significant decrease in FRET signal at 1 µg/mL (Fig. S1a). Using this concentration as baseline, subsequent tests with insoluble tau fractions from the entorhinal cortex (*n* = 4) or hippocampus (*n* = 5) of AD patients revealed that antibody C induced a statistically significant inhibitory effect on tau seeding (Fig. 1b and c, and table S2). With the entorhinal cortex, both antibodies targeting the MTBR, C and D, inhibited tau seeding, with antibody C showing superior effects (Fig. 1b). With the hippocampus, only antibody C was effective (Fig. 1c). Further analysis by Braak stages showed that only antibody C significantly reduced tau seeding in both Braak 3–4 (Fig. S1b) and Braak 5–6 (Fig. S1c). These results indicate that the anti-tau antibody targeting acK280 on MTBR was most potent in inhibiting tau seeding from AD brain extracts.

**Fig. 1:**
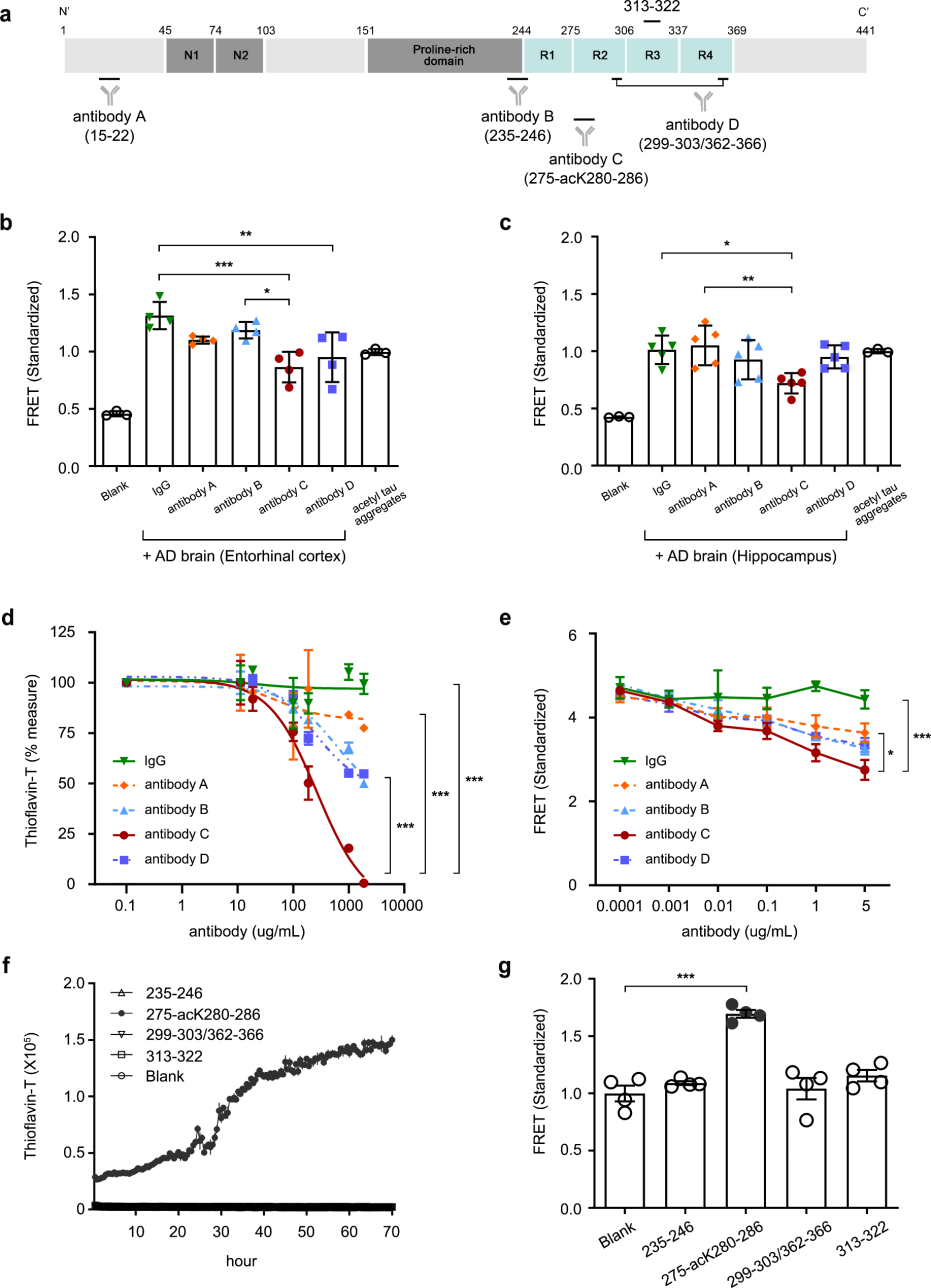
**a** A schematic domain map of tau 2N4R isoform and target epitopes of various anti-tau antibodies and epitope peptides. Relative location on the tau isoform of antibodies’ epitope sequences is represented by the antibody’s name and amino acid residue numbers within brackets **b**,** c** FRET signal of human Alzheimer’s disease insoluble tau fraction extract co-incubated with various anti-tau antibodies (1 µg/mL) at endpoint. Tau-FRET cells were treated with entorhinal cortical (*n* = 4) (**b**) or hippocampal (*n* = 5) (**c**) extract from Alzheimer’s disease patients and various anti-tau antibodies **d** ThT signal of acetylated tau aggregates co-incubated with various anti-tau antibodies at endpoint. Acetylated tau aggregates were incubated with ThT fluorescent dyes (1:1 ratio) and anti-tau antibodies at various concentrations for 70 h **e** FRET signal of acetylated tau aggregates co-incubated with various anti-tau antibodies at endpoint. Tau-FRET cells were treated with acetylated tau aggregates and anti-tau antibodies at various concentrations **f** ThT fluorescence signal of peptides corresponding to target epitope sequences of anti-tau antibodies. Each peptide was incubated with ThT fluorescent dyes (1:1 ratio) **g** FRET signal of peptides corresponding to target epitope sequences of anti-tau antibodies. Tau-FRET cells were treated with peptides corresponding to target epitope sequences of anti-tau antibodies at endpoint Two-way ANOVAs (**d**, **e**) and one-way ANOVAs were used for statistical analysis followed by Tukey’s multiple comparisons test. Line graphs present the mean ± SE determined from independent experiments represented by dots, each performed in triplicate. *p < 0.05, **p < 0.01, ***p < 0.001

Since tau acetylation is proposed to contribute to accelerated tau aggregation and AD pathology [7] and showed similar FRET signal to AD brain extracts (Fig. 1c), acetylated full-length tau (acTau) was used to illustrate the differing effect of anti-tau antibodies on tau seeding of AD brain extracts, their effect on acTau seeding and aggregation was evaluated with FRET assay and Thioflavin T (ThT) assay. While anti-tau antibodies reduced ThT signal in dose-dependent manner, antibody C showed the greatest inhibition of tau aggregation, reaching near to full removal of amyloid formation, albeit at high concentration of 1000 µg/mL (Fig. 1d). Additionally, tau seeding showed a treatment dose-dependent decrease by anti-tau antibodies. The reduction in tau seeding with antibody C was significantly greater compared to the other antibodies, with increasing treatment concentration (Fig. 1e).

As various sized tau fragments exist in brains and ISF among which few may constitute key tau pathogen [6], we speculated differing effects of antibodies inhibiting tau seeding could be derived from each antibody’s ability to target minimal tau fragments acting as seeding catalyst. We hence generated tau epitope peptides for each anti-tau antibodies targeting near or MTBR itself (Fig. 1a and table S1) to compare the tau aggregation or seeding potency of target epitopes. Aggregation of each epitope peptides was induced with addition of heparin and monitored by ThT assay. Only 275-acK280-286 exhibited an accelerated aggregation curve on ThT assay (Fig. 1f) and FRET intensities were significantly increased in cells treated with 275-acK280-286 (Fig. 1g), suggesting it is the most aggregation-prone and seed-competent species among the epitope peptides tested. Since the target sequence of antibody D, HVPGG, is relatively shorter than other peptides tested, longer tau peptides 295–311 and 358–372 were generated with HVPGG positioned in the middle (Fig. S2a) but these also showed little amyloid formation (Fig. S2b) and seeding (Fig. S2c). Also, as MTBR forms the core of tau aggregates in tauopathies [8] and MTBR fragments were recently detected in patient CSF [9], these fragments might represent the extracellularly released seed-competent tau species, potential targets of therapeutic antibodies (Fig. S2a). We hence investigated whether the MTBR fragments found in tauopathy CSF could aggregate or induce tau seeding. However, MTBR peptides did not induce amyloid formation (Fig. S2d) and seeding (Fig. S2e). These results show that peptide containing acetylated lysine-280 yield highest propensity for aggregation and seeding among the tested tau fragments, suggesting as an appealing target to remove via immunotherapy.

While the antibodies used are not from the exact same batch as those used in clinical trials and may exhibit differences in characteristics such as affinity, the direct comparison of the antigens targeted by the different antibodies (Fig. 1f, g) still supports our conclusion that acK280 is a more efficient target compared to others. MTBR forms a critical component of the β-sheet core of tau tangles [8] and contains the amyloid-forming motifs VQIINK and VQIVYK [10]. Our results also suggest that MTBR antibodies are more effective at inhibiting tau seeding and aggregation than N-terminus antibody, aligning with recent development trends focused on targeting MTBR. The P-G-G-G motif regulates tau aggregation by engaging in β-turn interactions with adjacent VQIINK and VQIVYK motifs, and its perturbation, such as via lysine acetylation, can lead to formation of seed-competent monomers [10]. Lysine deletion or acetylation may neutralize the positive charge within this region, yielding pathogenic neurodegenerative phenotypes [11]. Acetylation of K280, the lysine of VQIINK motif located in the second repeat, plays a key role in tau secretion and propagation. Its inhibition by immunotherapy ameliorated cognitive impairment and tau pathology in tau transgenic mice [12], further validating toxicity of this region. Limitations remain in identifying the exact tau fragments and PTMs that are key pathogens in AD brain ISF. While antibodies targeting the phosphorylated tau, which is also key pathologic tau PTM, are not included in this study [13] as well as preclinical antibodies targeting other acetylation sites [14], this study suggests that targeting acK280 in the MTBR region presents a promising approach among the latest clinical trial-stage antibodies tested. Future studies could explore comparative efficacies using additional antibodies, possibly in the context of targeting diverse PTM profile of tau pathologies [1].

## Electronic supplementary material

Below is the link to the electronic supplementary material.

Supplementary Material 1

## Data Availability

The datasets used and/or analyzed during the current study are available from the corresponding author on reasonable request.

## Abbreviations

PTMs: Post-translational modifications
Aβ: Amyloid beta
CSF: Cerebrospinal fluid
MTBR: Microtubule-binding region
ISF: Interstitial fluid
FRET: Fluorescence resonance energy transfer
AcTau: Acetylated full-length tau
ThT: Thioflavin T
