# Supplementary material for: Comparing anti-tau antibodies under clinical trials and their epitopes on tau pathologies
Source: Mol Neurodegener. 2024 Oct 19;19:76. doi: 10.1186/s13024-024-00769-x (PMC11490998; doi:10.1186/s13024-024-00769-x)
Supplement: Supplementary file 1 — Supplementary Material 1 [file 13024_2024_769_MOESM1_ESM.docx]

**Comparing anti-tau antibodies under clinical trials and their epitopes on tau pathologies**

Ha-Lim Song,^1,†^ Min-Seok Kim,^1,†^ Woo-Young Cho,^2^ Ye-Seul Yoo,^2^ Jae-You Kim,^2^ Tae-Wook Kim,^2^ Hyori Kim,^3^ Dong-Hou Kim,^2^ and Seung-Yong Yoon^1-2,4,*^

**†These authors contributed equally to this work.**

**Supplementary Figures**

**
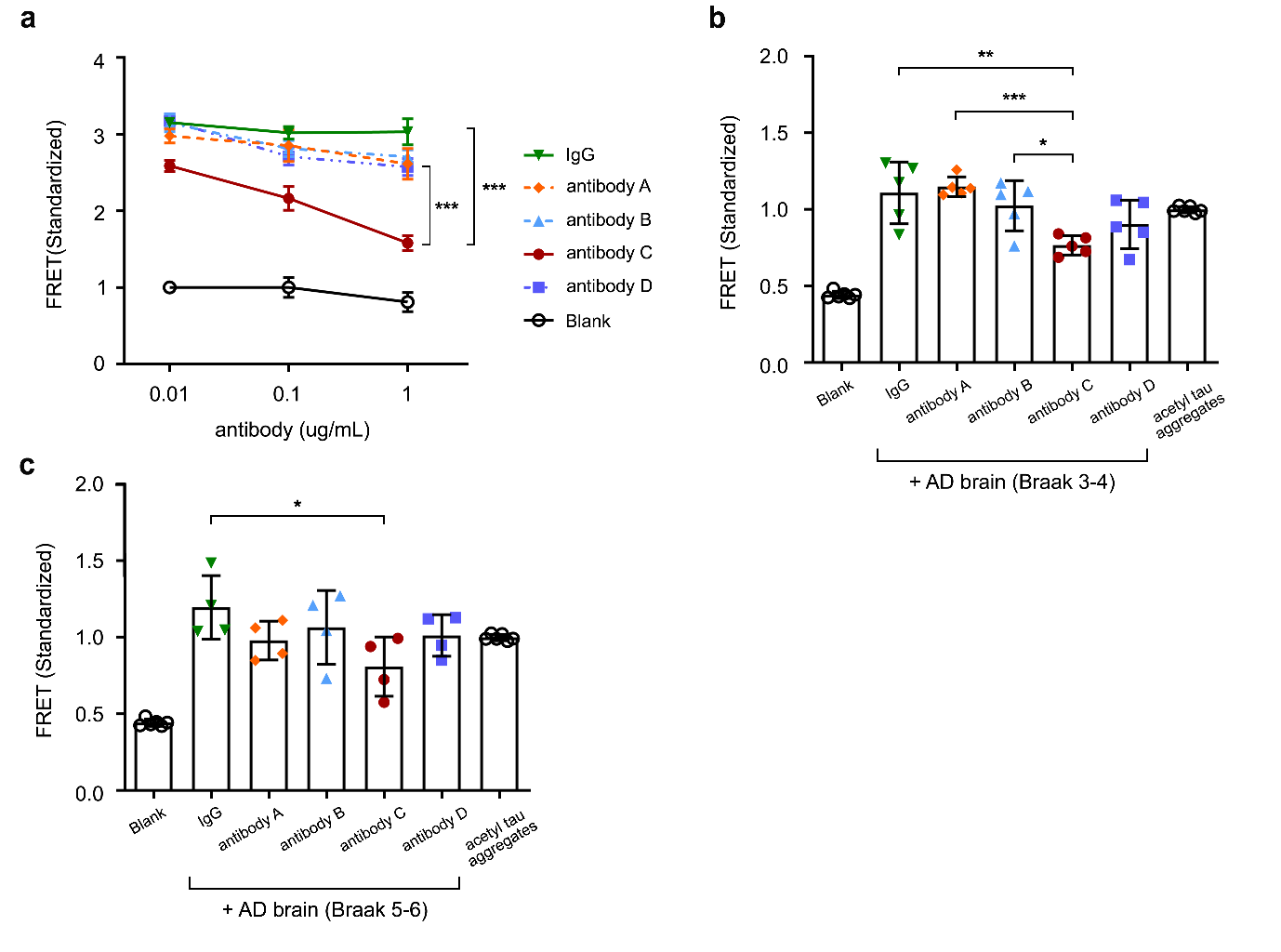
**

**Supplementary figure 1.**

**a** FRET signal of Alzheimer’s disease insoluble tau fraction extract co-incubated with various anti-tau antibodies at endpoint. Tau-FRET cells were treated with insoluble tau fraction from Alzheimer’s disease patient’s postmortem brain extract and anti-tau antibodies at various concentrations.

**b, c** Tau-FRET cells were treated with insoluble tau fraction from AD patients’ postmortem brain extracts from Braak stage 3-4 (*n* = 5) (**b**) or 5-6 (*n* = 4) (**c**).

Two-way ANOVA (a) and one-way ANOVAs were used for statistical analysis followed by Tukey’s multiple comparisons test. All assays were performed in triplicate, and the mean ± SE was determined from independent experiments represented by dots. Acetylated tau aggregates is used as positive control of tau seeding. **p* < 0.05, ***p* < 0.01, ****p* < 0.001.


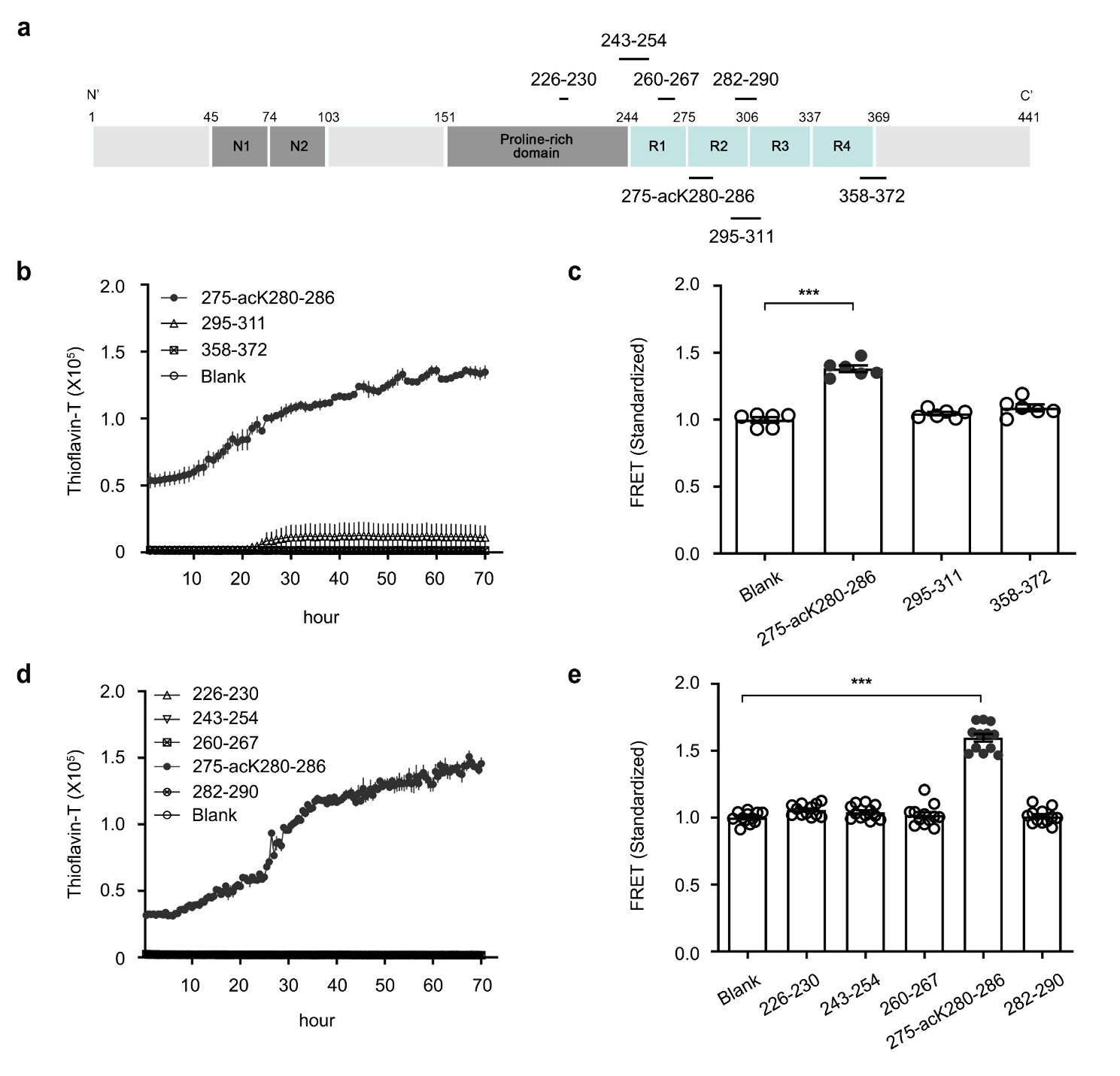


**Supplementary figure 2.**

**a** A schematic domain map of tau 2N4R isoform and MTBR fragments. Relative location on the tau isoform of each peptide sequence is represented by a bar accompanied by the number of amino acids sequences.

**b** ThT signal of peptides containing HPVGG. Peptides were incubated with ThT fluorescent dyes (1:1 ratio) for 70 hours.

**c** FRET signal of Tau-FRET cells treated with peptides containing HPVGG after 48 hours of incubation.

**d** ThT signal of MTBR fragments. MTBR fragments were incubated with ThT fluorescent dyes (1:1 ratio) for 70 hours.

**e** FRET signal of Tau-FRET cells treated with MTBR fragments after 48 hours of incubation.

One-way ANOVAs were used for statistical analysis followed by Tukey’s multiple comparisons test. All assays were performed in triplicate, and the mean ± SE was determined from independent experiments represented by dots. ****p* < 0.001 compared to Blank.

**MATERIALS AND METHODS**

**Generation of tau peptides**

The peptides were synthesized by Peptron (Osong, Republic of Korea). Information on the peptides used in the study is summarized in Supplemental Table 1. Lyophilized peptides were dissolved at 10 mg/mL in water or 100 mg/mL in DMSO. The peptide was purified by Shimadzu Prominence HPLC system (Purity >95%) with a 5-70% linear gradient of acetonitrile in 0.1% Trifluoroacetic acid.

**Monoclonal antibody purification**

Antibody C was generated from the stably expressing CHO cells. For antibodies A, B, and D, the genes encoding both the light and heavy chains of the antibodies were chemically synthesized (Thermo Fisher Scientific Inc., MA, USA) based on sequences from patents US 2016/0289309 A1, WO 17005734 A1, and WO 2019/077500 A1, respectively. For the light chain, the sequence includes the leader sequence of the human immunoglobulin kappa chain, variable light chain, and the human constant kappa domain. The heavy chain consists of a leader sequence, variable heavy chain, and the constant heavy chain derived from human IgG. The genes were sub-cloned into dual promoter mammalian expression vector. HEK293F cells (FreeStyle™ 293-F Cells, Invitrogen) were cultured in FreeStyle™ 293 Expression Medium (Invitrogen) containing penicillin and streptomycin (100 units/ml and 100 μg/ml, respectively) at 37°C in 7% CO2 on an orbital shaking incubator (135 rpm). The expression vector was transfected into HEK293F cells using 25-kDa linear polyethylenimine (Polysciences, Warrington, PA, USA). The mixture of 2 μg plasmid DNA and 4 μg linear polyethylenimine in 100 μl 150 mM NaCl solution was prepared for each milliliter of cell culture volume. Following 15 minutes incubation at room temperature, the mixture was added to HEK293F cells (2×10^6^ cells/ml) and the cells were grown for 5 days. The protein was purified from culture supernatant by AKTA Pure (GE Healthcare, Chicago, IL, USA) using MabSelect SURE column (GE Healthcare) per the manufacturer’s instructions. After purification, purified fractions were diluted in sample loading buffer (NuPAGE^®^ LDS Sample Buffer, Invitrogen) with reducing agent (NuPAGE^®^ Sample Reducing Agent, Invitrogen), and boiled for 5 min and subjected to SDS-PAGE (NuPAGE^®^ 4–12% Bis-Tris, Invitrogen) gel. The gel was stained by Coomassie Brilliant Blue R-250 (Amresco, Solon, OH, USA).

**Tau acetylation and aggregation**

For tau acetylation, 8 µM recombinant tau protein, 125 µM Acetyl CoA (Sigma-Aldrich, St. Louis, MO, USA), and 0.5 µg of purified GST-p300 were incubated for 3 hr at 30°C in acetylation buffer (10 mM HEPES, 50 mM NaCl, 1.5 mM MgCl_2_, 0.5 mM dithiothreitol (DTT), 2.5 mM EGTA, 0.1 mM EDTA) with agitation (Eppendorf Thermomixer C). Acetylated tau was initially reconstituted in 2 mM DTT for 1hr at RT. The mixture was subsequently incubated for aggregation with buffer (10 mM HEPES, 100 mM NaCl, 25 mM heparin, pH 7.4) with agitation at 700 rpm at 37°C.

**Human brain tissues**

Human brain samples from AD patients were obtained from the Human Brain Bank of Seoul National University (SNUHBB) and Korea Brain Bank Network (Supplemental Table 2). Pathological staging was provided according to Braak stages [1].

**Sarkosyl insoluble fraction**

Brain samples are dissociated in RAB buffer (100 mM MES (2-(N-morpholino)ethanesulfonic acid), 0.75 M NaCl, 1 mM EGTA, 0.5 mM MgSO4, 2 mM DTT, pH 6.8) with addition of protease and phosphatase inhibitors and incubated for 20 minutes on ice. Samples were centrifuged at 9,000 rpm for 20 minutes at 4°C. Pellet was resuspended in 1% sarkosyl buffer (1% sarkosyl, 10 mM Tris, 10% sucrose, 0.85 M NaCl, 1 mM EGTA, pH 7.4) then centrifuged at 13,000 rpm for 20 minutes at 4°C after 1 hour incubation at RT. Pellet was collected as sarkosyl insoluble fraction.

**Thioflavin T (ThT) assay**

For measurement of ThT fluorescence [2], 10-20 μM of tau protein or 25 μM various peptides were incubated with heparin [3] (Sigma-Aldrich) and 1 mM DTT in a 100 mM sodium acetate buffer (pH 7.0) in a 384-well clear bottom plate. Equimolar concentration ThT solution were added in triplicate with or without antibodies. ThT fluorescence was measured every 15 min at 37°C using CLARIOstar (BMG Labtech, Ortenberg, Germany) with excitation filter of 450 nm and emission filter of 510 nm. Blank wells were treated with buffer and ThT from the experimental conditions.

**Fluorescence resonance energy transfer (FRET)**

HEK293-Tau RD P301S FRET Biosensor [4] (ATCC CRL-3275, produced by Diamond lab) cells were plated (~20,000 cells per well) into 96-well black plates and incubated overnight at 37°C and 5% CO_2_. For tau and sarkosyl fraction, cells were transduced with 3 μg/mL tau protein or 1 μg/mL sarkosyl insoluble tau fraction from human brain extract using lipofectamine for 48 hours in triplicate with or without antibodies. For peptide experiments, cells were transduced with 20-25 μM of aggregated peptide material using lipofectamine for 48 hours. Blank wells were treated with buffer from the experimental conditions. Fluorescence was measured using CLARIOstar (BMG Labtech) and then analyzed as previously described [5]. All FRET analyzes were performed at z' values between 0.5 and 1 and positive control used tau protein.

**Statistical analysis**

To compare the assay signals using different antibodies, or peptides, one-way or two-way ANOVA was used with Tukey’s test as a post-hoc analysis. All analysis and graphical displays are generated and derived from a non-linear regression model fitting using GraphPad Prism v.8. Results are presented as mean ± SEM and *p*-value lower than 0.05 was considered statistically significant.

**Supplemental Tables**

**(Table 1) Information of antibodies and peptides**

| **Seq number** | **Antibody** | **Sequence** | **Solubility** |
| --- | --- | --- | --- |
| 15-22 | Antibody A | AGTYGLGD | N/A |
| 235-246 | Antibody B [6] | SPSSAKSRLQTA | DW (10mg/ml) |
| 275-acK280-286 | Antibody C [7] | VQIINacKKLDLSN | DMSO (100mg/ml) |
| 299-303/362-366 | Antibody D [8] | HVPGG | DW (10mg/ml) |
| 313-322 | N/A [6] | VDLSKVTSKCGS | DW (10mg/ml) |
| 226-230 | N/A | VAVVR | DW (10mg/ml) |
| 243-254 | N/A [9] | LQTAPVPMPDLK | DW (10mg/ml) |
| 260-267 | N/A | IGSTENLK | DW (10mg/ml) |
| 282-290 | N/A | LDLSNVQSK | DW (10mg/ml) |
| 295-311 | N/A | DNIKHVPGGGSVQIVYK | DW (10mg/ml) |
| 358-372 | N/A | DNITHVPGGGNKKIE | DW (10mg/ml) |

**(Table 2) Information of the human samples**

| **Frozen tissue** | | | | | |
| --- | --- | --- | --- | --- | --- |
| **Case ID** | **Braak stage** | **Age (y)** | **Gender** | **Post-mortem delay (h)** | **Brain region** |
| AD1 (A1918) | III-IV | 94 | Female | 16.5 | HPC |
| AD2 (A1815) | III-IV | 93 | Female | 13 | HPC, EC |
| AD3 (A1933) | III-IV | 69 | Male | 8.8 | HPC, EC |
| AD4 (A1712) | V-VI | 75 | Female | 8 | HPC |
| AD5 (A1803) | V-VI | 80 | Male | 14.75 | EC |
| AD6 (A2007) | V-VI | 82 | Female | 18 | HPC, EC |
| AD7(KBBN03000375) | V-VI | 82 | Male | 5 | Temporal |

*HPC: hippocampus, EC: entorhinal cortex*

REFERENCE

1. Braak H, Alafuzoff I, Arzberger T, Kretzschmar H, Del Tredici K. Staging of Alzheimer disease-associated neurofibrillary pathology using paraffin sections and immunocytochemistry. Acta Neuropathol. 2006;112(4):389-404.

2. Nanavaty N, Lin L, Hinckley SH, Kuret J. Detection and Quantification Methods for Fibrillar Products of In Vitro Tau Aggregation Assays. Methods Mol Biol. 2017;1523:101-11.

3. Shammas SL, Garcia GA, Kumar S, Kjaergaard M, Horrocks MH, Shivji N, et al. A mechanistic model of tau amyloid aggregation based on direct observation of oligomers. Nature communications. 2015;6:7025.

4. Holmes BB, Furman JL, Mahan TE, Yamasaki TR, Mirbaha H, Eades WC, et al. Proteopathic tau seeding predicts tauopathy in vivo. Proceedings of the National Academy of Sciences of the United States of America. 2014;111(41):E4376-85.

5. Zhang JH, Chung TD, Oldenburg KR. A Simple Statistical Parameter for Use in Evaluation and Validation of High Throughput Screening Assays. J Biomol Screen. 1999;4(2):67-73.

6. Plotkin SS, Cashman NR. Passive immunotherapies targeting Abeta and tau in Alzheimer's disease. Neurobiol Dis. 2020;144:105010.

7. Song HL, Kim NY, Park J, Kim MI, Jeon YN, Lee SJ, et al. Monoclonal antibody Y01 prevents tauopathy progression induced by lysine 280-acetylated tau in cell and mouse models. The Journal of clinical investigation. 2023;133(8).

8. Roberts M, Sevastou I, Imaizumi Y, Mistry K, Talma S, Dey M, et al. Pre-clinical characterisation of E2814, a high-affinity antibody targeting the microtubule-binding repeat domain of tau for passive immunotherapy in Alzheimer's disease. Acta neuropathologica communications. 2020;8(1):13.

9. Dolan P, Tam SJ, Nijjar T, Holden MR, Tourino C, Elmaarouf A, et al. Microtubule binding region (MTBR)-specific antibody PRX005 prevents pathological tau progression via blockade of neuronal internalization: Prothena; 2021 [Available from: https://s201.q4cdn.com/351053094/files/doc_presentations/2021/03/1/MICROTUBULE-BINDING-REGION-(MTBR)-SPECIFIC-ANTIBODY-PRX005-PREVENTS-PATHOLOGICAL-TAU-PROGRESSION-VIA-BLOCKADE-OF-NEURONAL-INTERNALIZATION.pdf. Accessed 16 Aug 2024.
